# Design and Experiment of a Clamping-Drive Alternating Operation Piezoelectric Actuator

**DOI:** 10.3390/mi14030525

**Published:** 2023-02-24

**Authors:** Mengxin Sun, Zhenwei Cao, Lukai Zheng

**Affiliations:** Department of Mechanical Engineering, Nanjing Institute of Technology, Nanjing 211167, China

**Keywords:** piezoelectric actuator, backward motion, alternating step and single-step actuation modes

## Abstract

In recent years, piezoelectric actuators, represented by inertial and inchworm actuators, have been widely applied because of their high accuracy and excellent responsiveness. Despite the development of various piezoelectric actuators, there remain some flaws in this technology. The sticking point is that the piezoelectric actuators based on the friction driving principle are prone to unwanted backward motion when outputting stepping motion. It is thus urgent to explore solutions from the perspectives of principle and structure. In this paper, a clamping-drive alternating operation piezoelectric actuator is proposed, the two feet of which are driven by two piezoelectric stacks, respectively. Due to double-foot alternate drive guide movement, backward movement is prevented in theory. By adopting the double-layer stator structure, integrated processing and assembly are facilitated. Meanwhile, a double flexible hinge mechanism is installed in the stator to prevent the drive foot from being overturned due to ineffectiveness and premature wear. In addition, the stator is equipped with the corresponding preload mechanism and clamping device. After the cycle action mechanism of one cycle and four steps is expounded, a model is established in this study to further demonstrate the principle. With the prototype produced, a series of experiments are performed. In addition, the amplitude of actuation of the stator is tested through amplitude experiment. The performance of the stator is evaluated by conducting experiments in the alternating step and single step actuation modes. Finally, the test results are analyzed to conclude that the actuator operating in either of these two modes can meet the practical needs of macro and micro actuation.

## 1. Introduction

At present, precision positioning devices have been increasingly applied in various fields such as precision machining, optical fiber docking and medical devices etc. [1,2,3,4,5,6]. Due to the development of advanced technology, there are more demanding requirements placed on precision actuators that attract widespread attention from researchers due to various advantages such as high precision, fast response, compact structure and self-locking [7,8,9,10]. In order to address the disadvantages of piezoelectric ceramic materials in terms of stroke, the drive foot is usually used by piezoelectric actuators to generate elliptical or rectangular motion tracks. Furthermore, it relies on friction to drive the actuator to achieve both large stroke and high precision motion. Currently, the piezoelectric motors reliant on friction can be divided into ultrasonic type [11,12,13], inertial type [14,15,16] and inchworm type [17,18,19].

Ultrasonic motor usually needs to work at a fixed frequency, and can achieve high-precision motion through position control system. Tan et al. designed a USM controller to meet the requirements of precision surgery. Based on the establishment and identification of the USM model, a proportional–integral–derivative (PID) controller is used as the main tracking controller with the parameters derived optimally using an linear–quadratic regulator (LQR)-assisted tuning approach. The experiment verifies the effectiveness of the compound controller [20]. Sarah Makarem et al. used data-driven technology to perform iterative feedback tuning of the PID controller of the ultrasonic motor. The new driving method is proved to be robust and less parameter dependent by using appropriate search algorithm, and the integral position error generated by the new driving method is far less than that of the single-source driving method [21]. Compared with the ultrasonic motor which needs to work in the ultrasonic frequency band, inertia and inchworm actuators can output large displacement at low frequency (less than 1000 Hz) [22,23]. Based on the stick–slip principle, inertial actuators usually release sawtooth wave signal onto a single piezoelectric ceramic to move. Sun et al. developed an inertial actuator with thin and thick compliant mechanism fitted at both ends, confirming the different stepping characteristics at both ends. After discussing the slight difference between forward and backward speeds, they improved the consistency of output performance [24]. Pinskier et al. conducted a study on a flexible stick–slip piezoelectric actuator which is small in size and simple to produce. It is capable to compensate for errors and achieve linear motion through model prediction control [25]. Based on stick–slip principle, this actuator features simple structure and flexible design. So far, there have been many prototypes developed by researchers. However, the working principle of relying on inertia makes backward motion inevitable during the actual test. The occurrence of backward motion destabilizes the actuator and affects the accuracy of positioning. The piezoelectric actuator based on the inchworm principle can be used to output large thrust. Ling et al. explored the asymmetric driving mode, clamping arrangement and simplified excitation signal sequence of a push-type inchworm piezoelectric actuator, so as to develop a lever mechanism purposed to amplify the step length. In addition, a hexagon output shaft was used to enhance the preload and bearing capacity. The simplified signal is effective in improving the output speed [26]. Ma et al. proposed a compact inchworm actuator driven by the deformation of two multi-functional driving feet. With four steps involved in each cycle, both stable step characteristics and high output force are obtained through stiffness optimization [27]. It is usually a complex process to achieve the timing control of inchworm piezoelectric actuator, and uncertainty can arise from the operation and locking of clamping, which contributes to the occurrence of backward motion.

**Figure 1:**
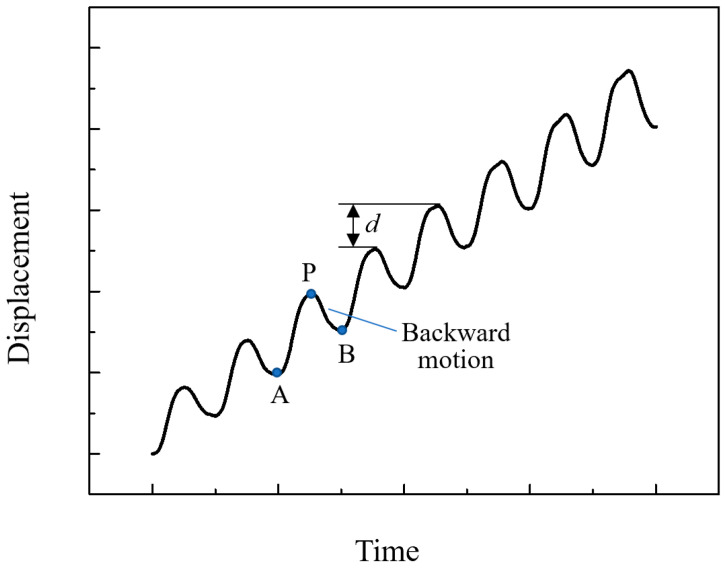
Backward motion of the actuator in step test.

In the previous stick–slip piezoelectric actuator, we determined that when the driving frequency is 60–100 Hz or higher, the displacement curve of the actuator output is relatively stable. When the driving frequency is further reduced (below 10 Hz) to obtain a smaller actuating step, as shown in the Figure 1, the step has obvious backward motion. This situation has also been described in other research papers. In most cases, d in the figure is defined as step distance. For precision positioning, however, the existence of backward motion restricts the realization of higher accuracy. It can be seen from the analysis of the above figure that in a cycle of movement, the AP segment is the driving foot that moves forward in the rising signal. Before reaching the peak P, there is a deceleration phenomenon. At this time, the signal has been converted from the rising segment to the falling segment. The guide stops gradually in the inertia until the PB segment moves backward.

In order to solve this problem encountered by piezoelectric actuators, researchers have carried out study from various perspectives such as structural design, principle exploration, signal optimization and so on [28,29]. Yang et al. proposed to balance the reverse friction in piezoelectric actuators through the forward friction caused by the elastic recovery of curved flexure hinges. Experimental results show that backward motion can be effectively reduced by this method [30]. Fan et al. put forward a synergic motion principle, which is effective in restricting the contact force in the driving process. Also, the backward motion is actively suppressed by the synergic drive of piezoelectric reactor. According to the experimental results, the actuator outperforms the previous ones [31]. Qin et al. designed a composite flexible hinge actively control the contact force between the driving foot and the slider of a stick-slip piezo-driven linear actuator. Active control is adopted to overcome the two major problems of backward movement and low load capacity. The prototype experiment shows that the load of the actuator is effectively improved under the active control of contact force [32]. In view of the backward motion of inertial actuators, Ding et al. proposed to prevent rollback by means of asymmetric clamping and specific phase coordination. Simulation and experimental results show that the prototype is applicable to ensure that no rollback occurs during each two-step cycle [33]. Jia et al. proposed a screwed type piezoelectric actuator with a hollow tube stator and rotor to directly drive a focusing lens to achieve a variation in the focal length. The electromechanical coupling model is established using the transfer matrix method, and the prototype is made to verify the correctness of the model. This kind of actuator uses thread self-locking to suppress the generation of backward motion, which can achieve high-precision linear positioning and large thrust-weight ratio actuation [34]. Burhanettin Koc et al. proposed a kinetic model to describe the condition under which slippage can occur between a slider and a stator. They also presented the structure and characteristics of a two phase inertia-drive-type piezoelectric motor, on which the proposed model was evaluated. As one actuator expands and the other shrinks, their respective hysteretic nonlinearities are canceled. The method alleviates the backward movement to a certain extent [35]. To sum up, the above studies have achieved the goal of reducing the backward motion of moving actuator to a certain extent. However, in case of high-precision stepping, it is still difficult to prevent the actuator from backward motion. Therefore, it is imperative to carry out further research on the optimization of actuator from the perspectives of principle and structure.

In order to resolve the significant backward motion of existing high-precision piezoelectric actuators, the clamping-drive alternating operation actuator principle is proposed in this study based on the inertia and inchworm driving principle. Besides, the double flexible hinge mechanism is adopted to ensure the sufficient contact of driving feet. With the development of a double-foot and double flexible hinge piezoelectric linear actuator, two modes, namely alternating step stability and single step high resolution precision action, are achieved to meet the needs of precise positioning.

## 2. Structure and Principle

### 2.1. Integral Actuator and Stator Structure

**Figure 2:**
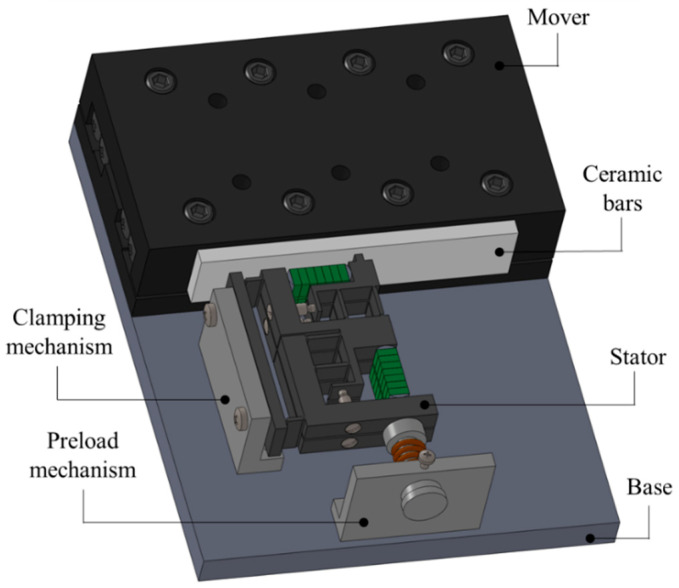
Structure of clamping-drive alternating operation piezoelectric actuator.

**Figure 3:**
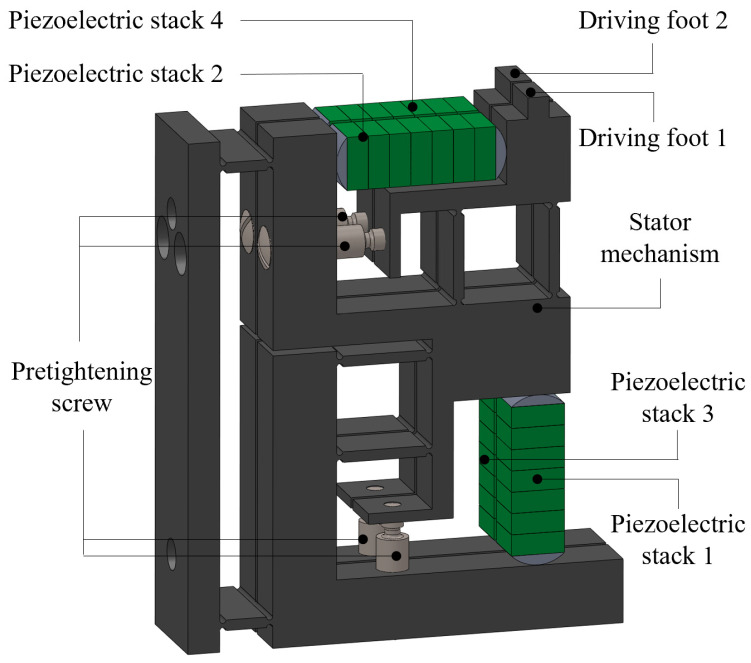
Structure of double flexible hinge stator.

A double-flexible hinged laminated double-foot piezoelectric linear motor is developed, the structure of which is shown in the Figure 1. As can be seen from this Figure 2, it consists mainly of a laminated stator, a clamping mechanism, a preload mechanism and a moving guide. The overall structure of the laminated stator is shown in the Figure 3. There are two piezoelectric stacks in each stator. By using preload screws, preload force is applied to the piezoelectric ceramics with semi-circular pads at both ends. A double flexible hinge guide mechanism is designed to be positioned in the transverse and longitudinal parts of the stator structure. Through collaboration between the clamping mechanism and the preload mechanism, it is ensured that the stator and the actuator are in sufficient contact. When there are specific signals inputted into the piezoelectric stacks, the two groups of piezoelectric stacks on the stator structure of each layer generate transverse and longitudinal vibrations respectively on the top of the driving foot, thus forming a closed motion path. Relying on friction force, the guide is driven to move in a straight line.

**Figure 4:**
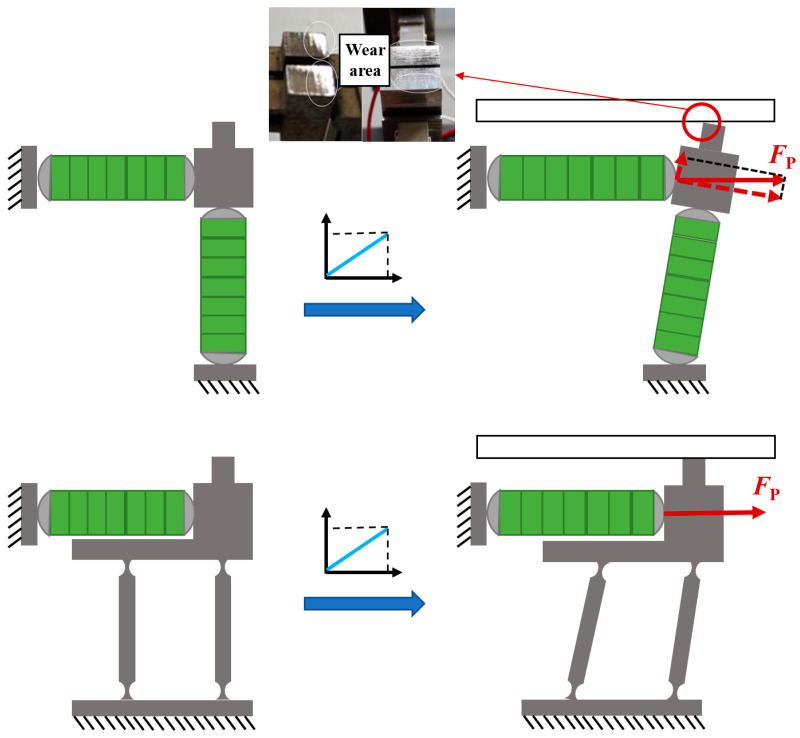
Kinematic comparison of guide mechanism with and without double flexible hinges.

The driving foot of the stator can be displaced in the horizontal and longitudinal directions respectively. According to the comparative analysis shown in the Figure 4, the friction caused by the driving foot on the guide acts in theory along the horizontal direction after the double flexible hinge guide is adopted, which increases the power required by the actuator and reduces wear effectively. The pre-pressure exerted in the longitudinal direction decreases the component along the horizontal direction. To a certain extent, slipping is avoided.

### 2.2. Preload Mechanism

**Figure 5:**
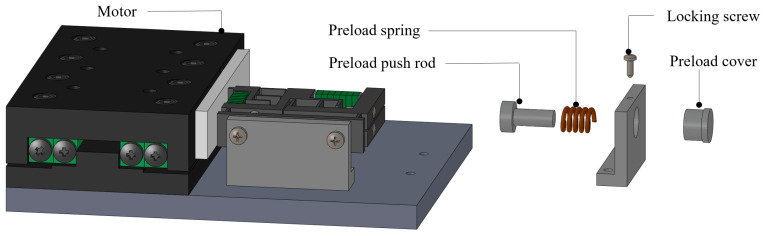
Structure of preload mechanism.

In order to realize the joint action of both feet, it is necessary to ensure that the contact state of both feet is consistent. On the one hand, integrated processing is adopted in the design to reduce the error, and on the other hand, repeated adjustment is required in the assembly to achieve the best state. The installation method of adopting rigid preloading mechanism has defects in ensuring that both feet contact the guide at the same time. It is difficult to further adjust the preload during assembly, and it is difficult to ensure simultaneous contact of both feet. So the flexible connection preloading mechanism is used to replace the original rigid connection mechanism. It is found that the flexible connection preload mechanism can effectively achieve the adjustability of the preload state. By adjusting the length of the spring, it is easier to test the required preload state in assembly. According to the preload mechanism, the spring is adopted as the main body and the push rod is used in combination to assist pressing, as shown in the explosion diagram of the Figure 5. In addition to providing effective thrust, it also ensures a certain level of elasticity.

### 2.3. Principle of Actuator

**Figure 6:**
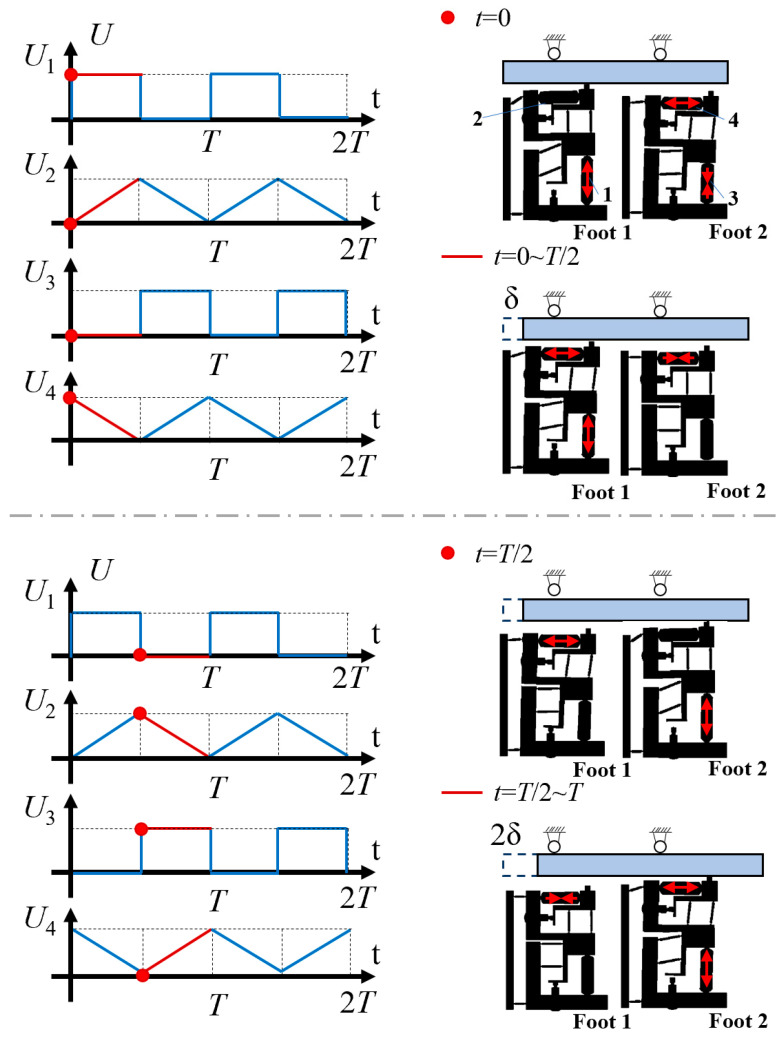
Piezoelectric stack signal and one-cycle motion process diagram.

Square wave and triangle wave signals are applied to four groups of piezoelectric stacks respectively, with the actuator used to output alternating step motion. According to the Figure 6, the motion of the actuator during a cycle can be classed into four typical states.

In the first stage (*t* = 0), the voltage applied on the piezoelectric stack 1 rises from 0 to *U*, which results in its rapid elongation to push the driving foot 1 against the guide. At the same time, the driving foot 2 is separated from the guide.

In the second stage (from *t* = 0 to *t* = *T*/2), the piezoelectric stack 2 is slightly extended due to the triangle wave signal in the ascending section, which causes the driving foot 1 to move a short distance *δ* because of friction force. At this time, the driving foot 2, which is driven by the piezoelectric stack 4, is in the return stage. In this stage, foot 1 is driving module and foot 2 is clamping module.

In the third stage (*t* = *T*/2), the voltage on the piezoelectric stack 3 rises from 0 to *U*, and the driving foot 2 comes into contact with the moving guide as a result of its rapid elongation. Due to voltage drop, the original length of the piezoelectric stack 1 is restored, and the driving foot 1 is separated from the guide.

In the fourth stage (from *t* = *T*/2 to *t* = *T*) the triangle wave signal extends the piezoelectric stack 4 slightly to promote the motion of the driving foot 2 under friction force, thus driving the actuator to move a short distance *δ*. At this time, the driving foot 1, which is driven by the piezoelectric stack 2, is in the return stage. In this stage, foot 1 is clamping module and foot 2 is driving module.

If the motion within a cycle is repeated, the actuator continues to move in a straight line, and the distance of motion within the cycle is 2*δ*.

## 3. Theoretical Analysis

**Figure 7:**
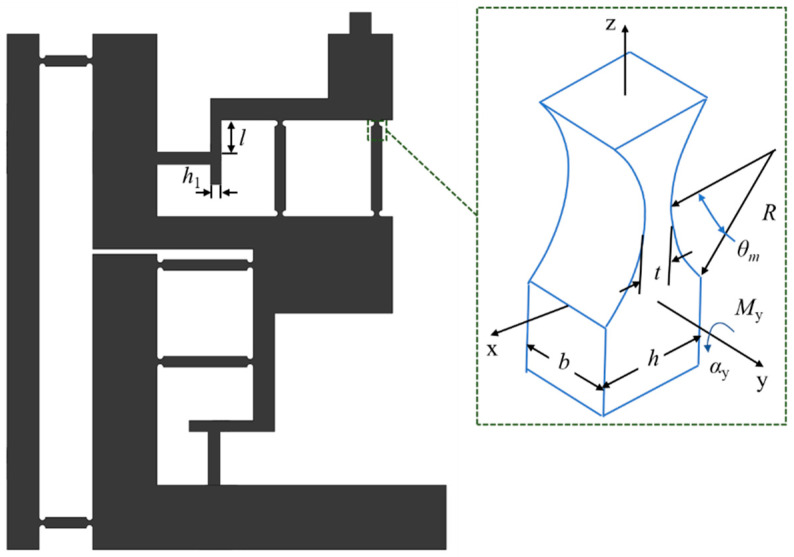
Structural parameters of transverse flexure hinge of stator.

The output performance of the actuator is largely determined by the stator. When the actuator is in stable motion, the motion speed of the actuator can reach the same level as its driving foot. The Figure 7 shows how the stator is structured, whose longitudinal motion requires only locking force and whose output is largely determined by the characteristics of transverse motion. Therefore, the key parameter relates to the structure of the transverse double flexible hinge.

As shown in the Figure 7, there circular flexural hinges at both ends of the double flexible hinge guiding mechanism, as shown on the right of the Figure 6, in which the structural parameters are marked. Where, *b* is the thickness of the flexure hinge, *t* is width of the beam connecting flexure hinge, *R* is radius of the semi-circle flexure hinge, *h* is the width of the flexure hinge, *h*_1_ is the width of the beam. There are four circular flexural hinges used, all of which are connected in series in pairs. Since these two groups are deployed in parallel with each other, the total stiffness is equal to the stiffness of a single circular flexural hinge. With straight circular flexural hinges adopted, *θ*_m_ = 90° and *q* = *R*/*t*. Thus, the stiffness of the double flexible hinge can be expressed as follows,

(1) $$\frac{1}{k_{f}}=\frac{\alpha_{y}}{M_{y}}=\frac{12}{EbR^{2}}\left[\frac{2q^{3}(6q^{2}+4q+1)}{(2q+1){\left(4q+1\right)}^{2}}+\frac{12q^{4}(2q+1)}{{\left(4q+1\right)}^{\frac{5}{2}}}\arctan(4q+1)\right]$$

where, *E* is Elastic modulus. The preloaded beam also exhibits stiffness to some extent, which can be expressed as follows:

(2) $$k_{b}=EI=Ebh_{1}{}^{3}$$

**Figure 8:**
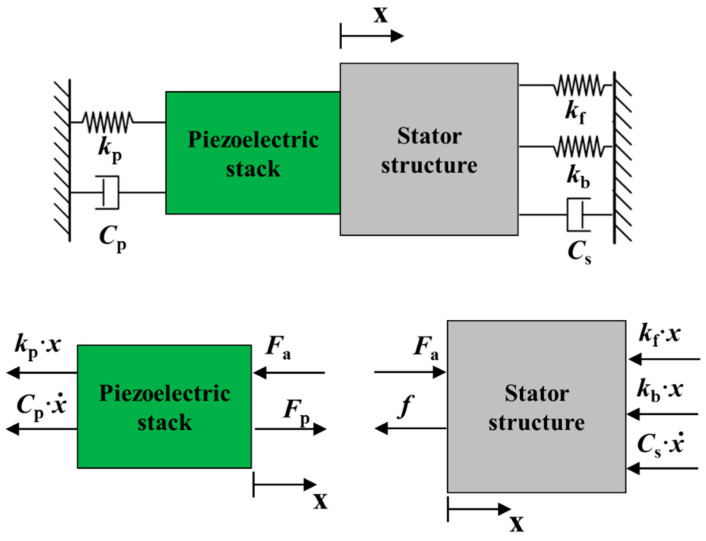
Dynamic model and force analysis of stator.

As shown in the Figure 8, the dynamic model of the single-layer stator can be simplified into that comprised of the stator structure and piezoelectric stack, including the stiffness damping system. Also, the stress in each part is indicated in the figure. Where, *k*_p_ is the Stiffness of the piezoelectric stack, *C*_p_ is the damping coefficients of the piezoelectric stack, *C*_s_ is the damping coefficients of the piezoelectric stack, *F*_a_ is the internal force of piezoelectric stack and stator structure, *F*_p_ is the output force of the piezoelectric stack, *f* is the friction force.

The system vibration equation can be written as,

(3) $$\left[\begin{array}{c} m_{p}\\ m_{s} \end{array}\right]\left[\begin{array}{cc} \ddot{x}(t) & \ddot{x}(t) \end{array}\right]+\left[\begin{array}{c} C_{p}\\ C_{s} \end{array}\right]\left[\begin{array}{cc} \dot{x}(t) & \dot{x}(t) \end{array}\right]+\left[\begin{array}{c} k_{p}\\ k_{f}+k_{b} \end{array}\right]\left[\begin{array}{cc} x(t) & x(t) \end{array}\right]=\left[\begin{array}{c} F_{p}-F_{a}\\ F_{a}-f \end{array}\right]$$

where, *m*_p_ is the mass of the piezoelectric stack, *m*_s_ is the mass of the stator structure. The lateral vibration equation can be expressed as,

(4) $$(m_{p}+m_{s})x(t)+(C_{p}+C_{s})x(t)+(k_{p}+k_{f}+k_{b})x(t)=F_{p}-f$$

The input voltage on the transversely arranged piezoelectric stack can be expressed as,

(5) $$\frac{U_{p}(s)}{U_{0}(s)}=k_{a}\frac{1}{R_{c}Cs+1}$$

where, *U*_0_(*s*) is the initial voltage, ka is the amplification ratio of input voltage for the piezoelectric stack, *R*_c_ is the resistance of the driving circuit, *C* is the capacitance of the piezoelectric stack. Based on the above formula, the relationship between the lateral output displacement *X*(*s*) and the initial voltage *U*_0_ under the Laplace transform can be obtained as follows,

(6) $$\frac{X(s)}{U_{0}(s)}=\frac{nd_{33}k_{p}k_{a}}{\left(R_{c}Cs+1)[(m_{p}+m_{s})s^{2}+(C_{p}+C_{s})s+(k_{p}+k_{f}+k_{b})\right]}$$

where, *n* is the number of layers of piezoelectric stack, *d*_33_ is the piezoelectric coefficient of the piezoelectric stack. According to the references, the value of *n* is 100, the values of resistance of the driving circuit and capacitance of piezo stack is about 10 Ω and 1.4 × 10^−6^ F. According to the above relationship, the displacement output of the stator can be simulated by using Matlab. When the structural parameters of the stator *b* = 2 mm, *R* = 0.5 mm, *t* = 0.5 mm, and *h*_1_ = 2 mm, apply the signal with voltage of 100 V and frequency of 1 Hz on the piezoelectric stack, and the transverse output displacement of the stator is 12.5 μm.

**Figure 9:**
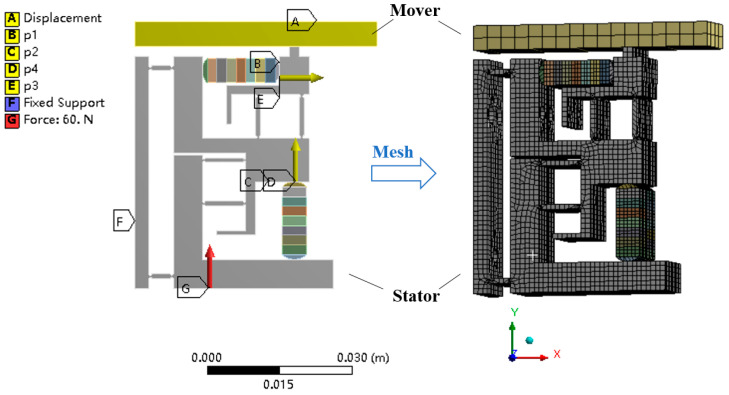
The model and mesh division of the actuator.

On the basis of the analysis of the stator step, the finite element simulation of the stator drive foot and the output motion of the mover is carried out. The initial conditions and mesh division are shown in the Figure 9. Hex dominant method is used in mesh when the body size is defined as 1 mm, and the element number is 20,716. In the simulation, the stator bottom is applied with a preload of 60 N, the left side is fixed, and the mover only retains the transverse displacement. The stator and mover are in frictional contact, and four piezoelectric stacks are applied with corresponding input signals. Because of the low actuation frequency, the quasi-static process is used for analysis.

**Figure 10:**
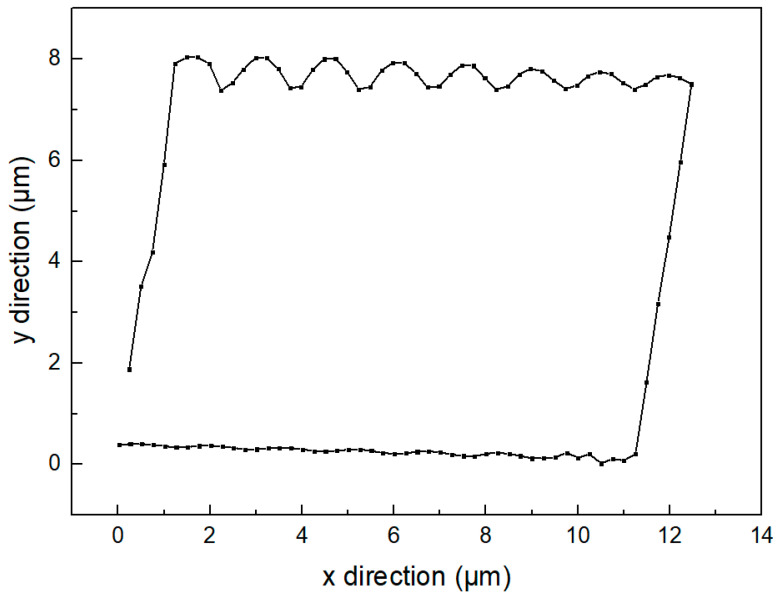
Motion trajectory of drive foot.

**Figure 11:**
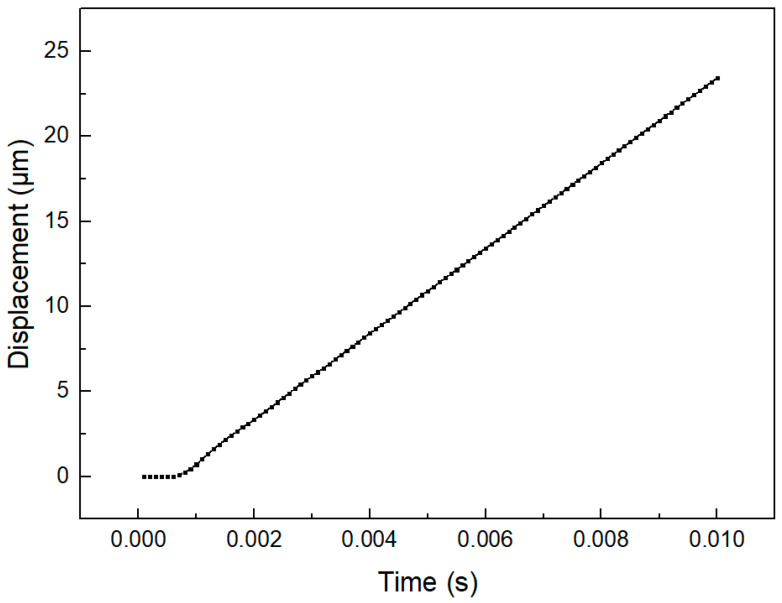
Displacement-time diagram of the mover.

The one-cycle motion track of the driving foot under the contact state between the stator and the mover can be obtained as shown in the Figure 10. It can be seen from the figure that the motion track of the single driving foot is a parallelogram. Due to the fluctuation of the displacement in the y direction, the motion step of a single foot does not fully affect the mover. As shown in the Figure 11, the moving distance of the mover is about 23.4 μm in a cycle.

## 4. Experimental Research

### 4.1. Prototype and Experimental Environment

**Figure 12:**
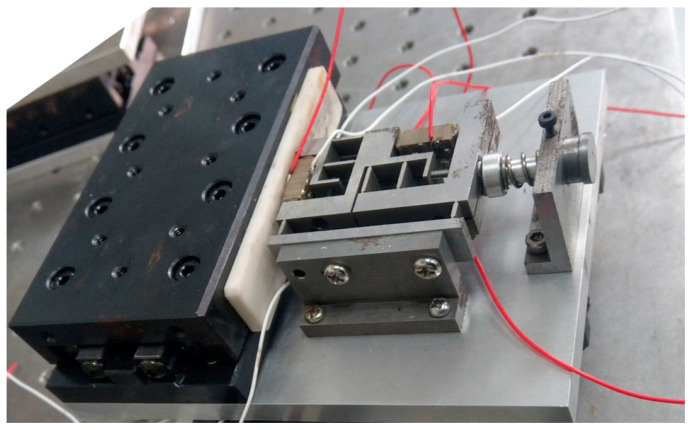
Piezoelectric linear motor prototype.

**Figure 13:**
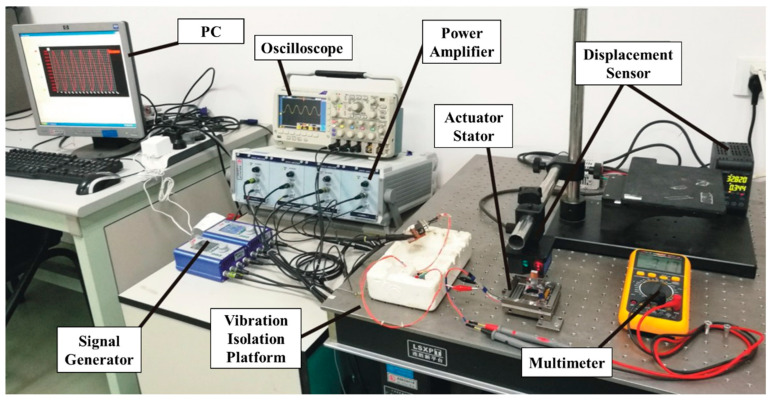
Experimental environment.

In order to verify the principle while demonstrating the advantages of the proposed mechanism, a prototype piezoelectric actuator with two feet and two flexible hinges was produced, as shown in the Figure 12. The double-layer stator fitted with flexible hinges was processed through the wire cutting method. Then, the experimental environment as shown in the Figure 13 was constructed. The four-channel square-triangle wave signals were generated by the signal generator and applied to the four groups of piezoelectric stacks respectively using the four-channel power amplifier. After the displacement output of the motor was measured by the laser displacement sensor, the output signals were displayed on the PC.

The piezoelectric stack used by the actuator is NAC2013-H14 piezoelectric stack of Noliac Company (Kvistgaard, Denmark). Its performance parameters are listed in Table 1.

### 4.2. Amplitude Test

**Figure 14:**
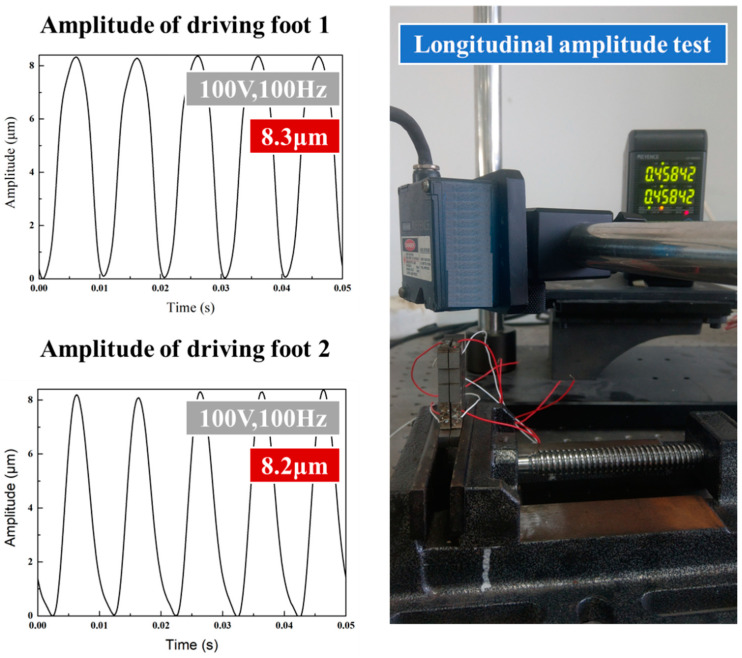
Longitudinal amplitude test diagram of double flexible hinge actuator drive foot.

**Figure 15:**
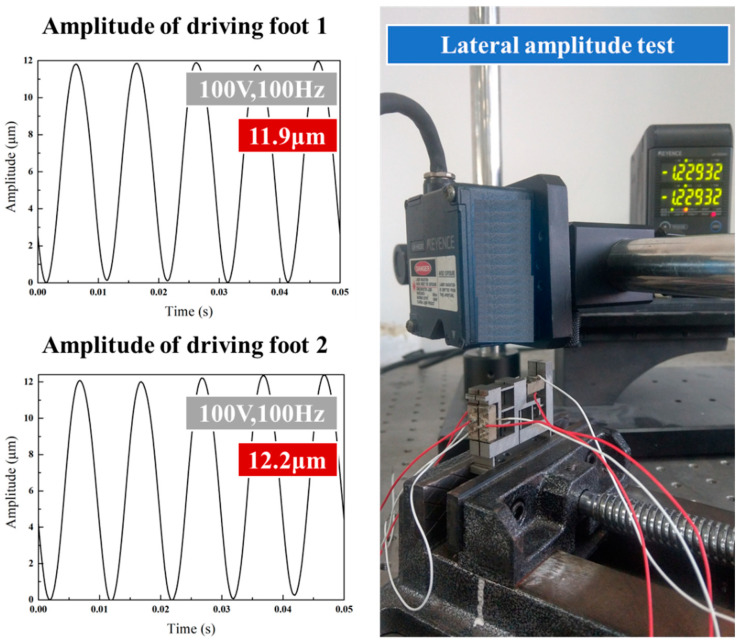
Transverse amplitude test diagram of double flexible hinge actuator drive foot.

Prior to the test on actuator performance, the stator was tested for amplitude, so as to determine the outcome of installing the piezoelectric stack in the stackable stator. With a sinusoidal voltage signal of 100 V 100 Hz applied to the stack, the transverse and longitudinal performances of the driving feet were evaluated using a laser displacement sensor. As shown in the Figure 14, the longitudinal amplitude of driving feet 1 and 2 is 8.3 μm and 8.2 μm on average, respectively. As also can be seen from the Figure 15, the average lateral amplitude of driving feet 1 and 2 is 11.9 μm and 12.2 μm, respectively. The transverse and longitudinal displacements are highly consistent with each other for the two driving feet.

### 4.3. Double-Foot Alternating Motion Mode

**Figure 16:**
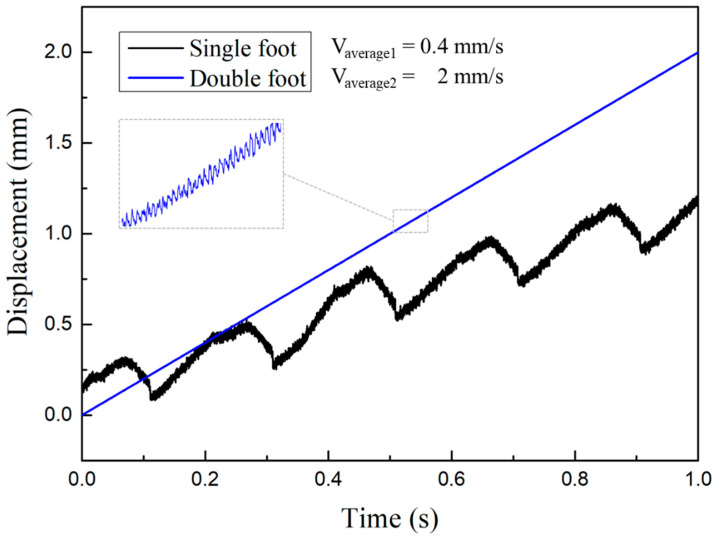
Comparison curve of displacement output of single foot and double foot drive.

When a certain level of prepressure was exerted, the single-foot and double-feet drive modes were tested respectively. When the input signal voltage reached 100 V, the curve diagram of the actuator output displacement which changed over time was measured, as shown in the Figure 16.

As shown in the Figure 16, the output speed of the actuator is 2 mm/s on average when the actuator is driven by two feet. In case of single foot drive, only half of a cycle is active. Despite an inertial effect, it still drives the guide back in reverse when the foot is driven back. At this time, the average speed of the actuator is 0.4 mm/s. It can also be seen from the figure that, the speed of a double-feet actuator is slightly higher than twice that of a single foot actuator.

By adjusting the frequency and voltage of the input signal, the actuator was further tested.

**Figure 17:**
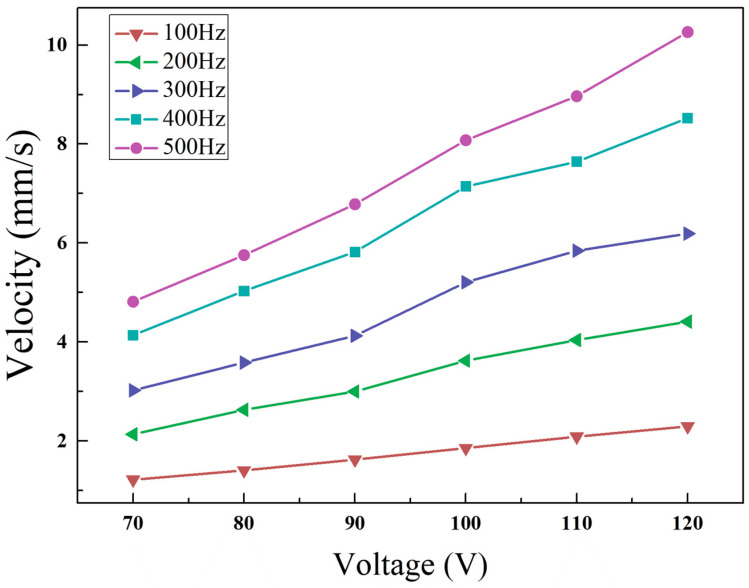
Piezoelectric actuator output performance curve.

As can be seen from the Figure 17, the speed of linear motor increases linearly with the rise of voltage. Given a driving signal of 120 V and 500 Hz, the motor can reach the maximum speed which is about 10.14 mm/s. According to the changes of frequency and voltage, a series of velocity curves of the actuator were fitted linearly to obtain the following velocity formula.

(7) v=(0.0002f+0.002)U−0.055f+0.31

**Figure 18:**
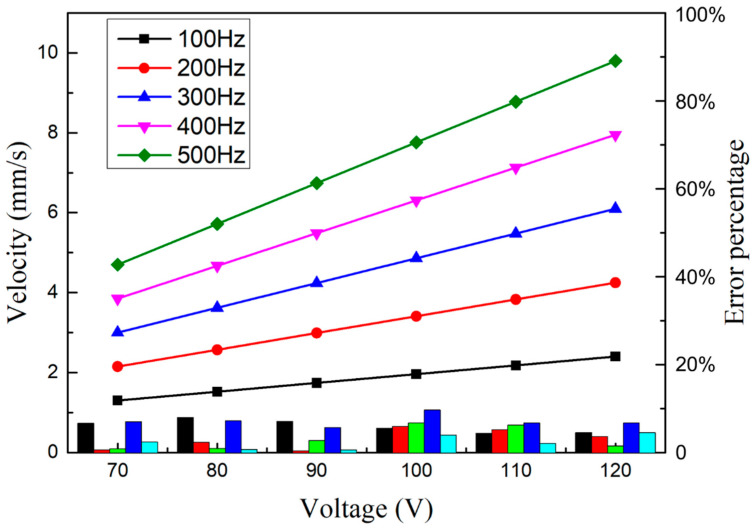
Fitting curve and error value.

To predict the output speed under the input condition, the corresponding voltage and frequency values can be substituted respectively, as shown in the Figure 18. The formula was used to compare the fitting data with the actual value, with the error obtained in percentage for each velocity point. It can be seen from the figure that the error of each velocity point under the context of linear fitting falls within 10%, mostly below 5%. Thus, the output performance of the actuator can be better described.

**Figure 19:**
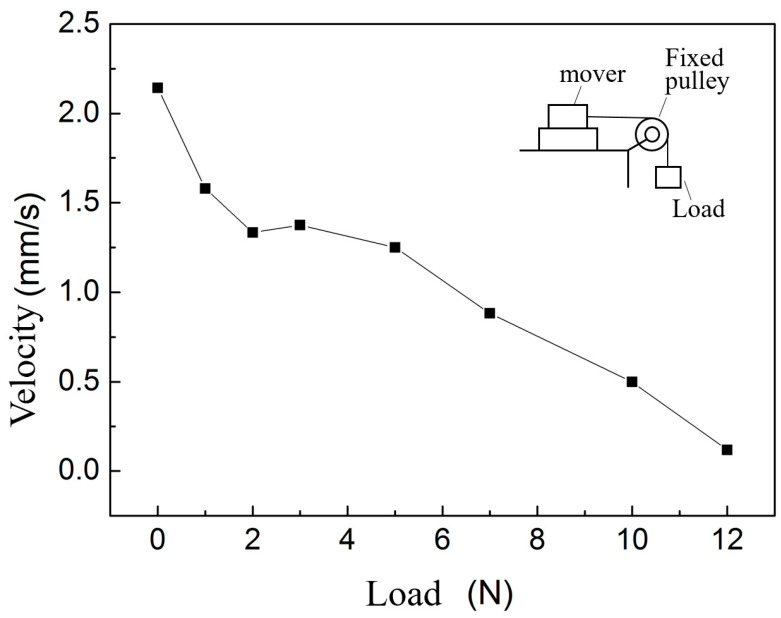
Load characteristic curve of the actuator.

Apply 100 V, 100 Hz square-triangle wave signal to the piezoelectric stack, and hang weights on one side of the guide to test the thrust of the motor. The load characteristic curve of the actuator is shown in the figure. It can be seen from the Figure 19 that the motor velocity decreases rapidly with the increase of load. The maximum thrust is about 12 N, and the load characteristics still need to be improved.

### 4.4. Single Step Actuation Mode

**Figure 20:**
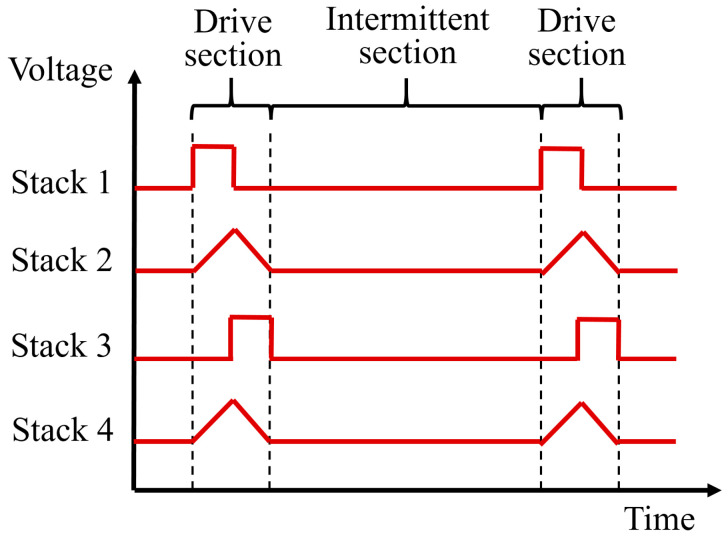
Input signal of single step actuation mode.

In order to test the actuator for its minimum output displacement, the single step actuation mode was adopted, as shown in the Figure 20. The input signal is referred to as the periodic signal of the drive section and the intermittent section, and the signal of the drive section is consistent with that of the alternating step mode. In the intermittent section, there is no signal inputted. Instead, the four output signals are respectively inputted into the corresponding piezoelectric stacks.

**Figure 21:**
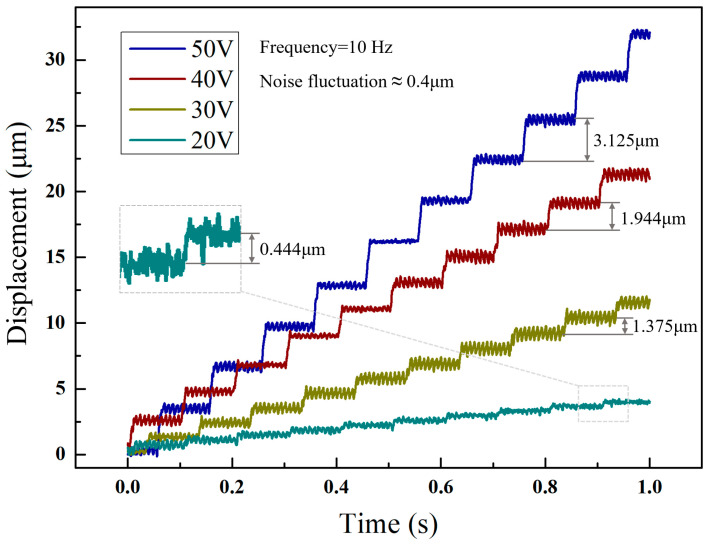
Displacement of actuator with different voltages under single step actuation mode.

By reducing the voltage and frequency, the minimum step of the actuator was obtained. The Figure 21 shows a series of resolution tests performed under the single step actuation mode. According to the Figure 16, the average motor step length is 3.125 μm, 1.944 μm, 1.375 μm and 0.444 μm when the drive signal frequency is 10 Hz and the voltage is 50 V, 40 V, 30 V and 20 V, respectively. When the voltage declines, it is difficult to identify the single step operation. Thus, the resolution of the motor was determined as roughly 0.444 μm.

### 4.5. Results Analysis

In view of the inconsistency between the results of alternating step mode and single step mode, the actuator can be analyzed under ideal conditions. When the actuator moves in the alternating step mode, the trajectory of the driving foot is rectangular, and the actuator driven by the friction force moves uniformly in a straight line. The displacement of the actuator movement can be simply expressed as follows,

(8) $$x(t)=2nd_{33}\beta U_{C}\frac{t}{T}$$

where, *β* is the structural stiffness coefficient, *U*_c_ is the maximum voltage on transverse piezoelectric stack. The corresponding velocity is as follows:

(9) $$\dot{x}(t)=2nd_{33}\beta U_{C}\frac{1}{T}$$

The step distance and the average thrust of both feet in a cycle can be calculated as follows,

(10) $$d_{a}=2nd_{33}\beta U_{C}$$

(11) $$\bar{F}=\frac{1}{T}\int_{0}^{T}\mu (nd_{33}\beta U_{D}+P)dt=\mu (nd_{33}\beta U_{D}+P)$$

where, *U_D_* is the maximum voltage on longitudinal piezoelectric stack, *P* is the preload force. However, the impact caused by inertia in the alternating actuation mode is ruled out in the single step actuation mode, and it is inappropriate to conduct the analysis solely under the ideal conditions.

At this time, the actuator may come into contact with the two driving feet simultaneously. It is assumed that the friction between the left driving foot and the actuator is *f*_1_(*t*), with the right driving foot in the return stage. If it is not completely separated from the actuator, the friction between the foot and the actuator is assumed to be *f*_2_(*t*). In this circumstance, the friction of the actuator guide itself is negligible, and for the actuator it can be known that,

(12) $$M\ddot{x}(t)=f_{1}(t)-f_{2}(t)$$

where *M* represents the mass of the mover.

(13) $$\left\{ \begin{array}{l} f_{1}(t)=\mu (nd_{33}\beta U_{D}+P)\\ f_{2}(t)=\mu P \end{array}\right.$$

It can be known that the motor speed in a single step can be expressed as follows:

(14) $$\dot{x}(t)=\mu nd_{33}\beta U_{D}Mt/T$$

Apparently, the speed of the actuator increases continuously. The speed of uniform motion as obtained before is shown in Formula (14). Therefore, the motor speed reaches its maximum at

(15) $$t_{0}=2U_{C}/\mu U_{D}M$$

And the actuator begins to move at a uniform speed. On this basis, it is inferred that the distance of a single motor operating step within one cycle is as follows:

(16) $$d_{s}=\frac{\mu nd_{33}\beta U_{D}Mt_{0}{}^{2}}{2T}+2nd_{33}\beta U_{C}(T-t_{0})/T$$

After one cycle, the left and right driving feet revert to the initial state immediately because the signal returns to zero, which causes the actuator to retract.

As can be known by comparing the operating step distance of the actuator in alternating step actuating mode and that in single step actuating mode within a cycle,

(17) $$\frac{d_{a}}{d_{s}}=\frac{T}{T-\mu M}$$

The proportion parameter indicates the variation in accuracy between these two modes, which is related only to the period of the input signal, the friction coefficient between the stator and the stator and the quality of the stator. As for the precision ratio of the two modes, it can be adjusted by changing the above parameters, so as to meet the needs of practical applications.

## 5. Conclusions

The present study deals with the design, analysis and performance test of clamping and driving alternating double-foot piezoelectric linear actuator. A cascade stator structure with two legs is developed to achieve the function of clamping and driving foot in turn. Through time sequence control, each foot of the actuator can alternately drive it to move linearly, which is effective in preventing backward motion during the process of output motion. Especially, when the step distance tends to reach the micron level and the frequency tends to reach 10 Hz or lower, a clear step curve is still maintained, which facilitates control in subsequent applications. In the stator, double flexible hinges are used to guide the direction of each vibration. In this way, the driving foot can be prevented from deflection to a certain extent and one end of the contact surface of the driving foot can be protected against premature excessive wear, which avoids the deterioration in overall performance of the actuator. Through a series of experiments, the feasibility of the actuator is verified. The actuator exhibits good linearity, and can move in two modes, namely alternating step and single step, which meets the needs of macro and micro actuation in various scenarios.

## Figures and Tables

**Table 1:** Parameters of piezoelectric stack.

Parameter	Size (mm × mm × mm)	Free Stroke, Max (μm)	Piezoelectric Constant, d_33_ (10^−12^ C/N)	Capacitance (nF)	Stiffness (N/μm)
Value	5 × 5 × 14	19.8	443	1080	118

## Data Availability

All research data are in the paper.

## Nomenclature

*b*	Thickness of the flexure hinge
*t*	Width of the beam connecting flexure hinge
*R*	Diameter of the semi-circle flexure hinge
*h*	Width of the flexure hinge
*h*_1_	Width of the beam
*E*	Elastic modulus
*K*_p_	Stiffness of the piezoelectric stack
*C*_*p*_	Damping coefficients of the piezoelectric stack
*C*_*s*_	Damping coefficients of the piezoelectric stack
*F*_*a*_	Internal force of piezoelectric stack and stator structure
*F*_*p*_	Output force of the piezoelectric stack
*f*	Friction force
*m*_*p*_	Mass of the piezoelectric stack
*m*_*s*_	Mass of the stator structure
*U*_0_ *(s)*	Initial voltage
*k*_*a*_	Amplification ratio of input voltage for the piezoelectric stack
*Rc*	Resistance of the driving circuit
*C*	Capacitance of the piezoelectric stack
*X(s)*	Lateral output displacement
*n*	Number of layers of piezoelectric stack
*d*_*33*_	Piezoelectric coefficient of the piezoelectric stack
*β*	Structural stiffness coefficient
*Uc*	Maximum voltage on transverse piezoelectric stack
*d*_*a*_	Step distance
*U*_*D*_	Maximum voltage on longitudinal piezoelectric stack
*P*	Preload force
*M*	Mass of the mover
